# Effect of Peierls transition in armchair carbon nanotube on dynamical behaviour of encapsulated fullerene

**DOI:** 10.1186/1556-276X-6-216

**Published:** 2011-03-14

**Authors:** Nikolai A Poklonski, Sergey A Vyrko, Eugene F Kislyakov, Nguyen Ngoc Hieu, Oleg N Bubel', Andrei M Popov, Yurii E Lozovik, Andrey A Knizhnik, Irina V Lebedeva, Nguyen Ai Viet

**Affiliations:** 1 Physics Department, Belarusian State University, pr. Nezavisimosti 4, Minsk 220030, Belarus; 2 North Carolina Central University, Durham, NC, 27707, USA; 3 Institute of Spectroscopy, Fizicheskaya Str. 5, Troitsk, Moscow Region, Russia, 142190; 4 RRC "Kurchatov Institute", Kurchatov Sq. 1, Moscow, Russia, 123182; 5 Kintech Lab Ltd, Kurchatov Sq. 1, Moscow, Russia, 123182; 6 Moscow Institute of Physics and Technology, Institutskii pereulok 9, Dolgoprudny, Moscow Region, Russia, 141701; 7 Institute of Physics and Electronics, Hanoi, Vietnam

## Abstract

The changes of dynamical behaviour of a single fullerene molecule inside an armchair carbon nanotube caused by the structural Peierls transition in the nanotube are considered. The structures of the smallest C_20 _and Fe@C_20 _fullerenes are computed using the spin-polarized density functional theory. Significant changes of the barriers for motion along the nanotube axis and rotation of these fullerenes inside the (8,8) nanotube are found at the Peierls transition. It is shown that the coefficients of translational and rotational diffusions of these fullerenes inside the nanotube change by several orders of magnitude. The possibility of inverse orientational melting, i.e. with a decrease of temperature, for the systems under consideration is predicted.

## Introduction

The structure and elastic properties of carbon nanotubes are studied in connection with the perspectives of their applications in nanoelectronic and nanoelectromechanical devices and composite materials, and are also of fundamental interest, particularly for physics of phase transitions. For example, superconductivity [1], commensurate-incommensurate phase transition in double-walled nanotubes [2], spontaneous symmetry breaking with formation of corrugations along nanotube axis [3] and structural Peierls transition in armchair nanotubes [4-9] have been considered. In the present Letter, we consider a fundamentally new phenomenon related to phase transitions in nanosystems. In other words, we consider the possibility of inverse orientational melting for molecules encapsulated inside nanotubes caused by structural Peierls transition in the nanotubes.

**Figure 1:**
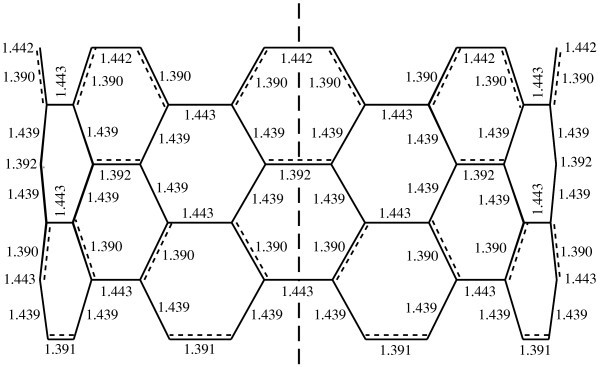
**Calculated Kekule structure corresponding to the ground state of the (8,8) nanotube**. Nanotube axis is shown by the dashed line.

The possibility of Peierls transition in carbon nanotubes was first considered in [4]. As a result of this transition, armchair nanotubes become semiconducting at low temperature, and Peierls distortions lead to the Kekule structure (see Figure 1) with two essentially different C-C bond lengths and a triple translational period (three times more hexagons in the translational unit cell). In previous studies, the Peierls gap [5-7] and the temperature of the transition to the metallic phase with equal C-C bond lengths [4,5,8] were estimated. Recently, the Kekule structure was calculated for the ground state of an infinite armchair (5,5) nanotube by PM3 semiempirical molecular orbital calculations [9]. It was shown that, for the (5,5) nanotube, the difference between C-C bond lengths for semiconducting phase is 0.03 Å, whereas the difference between nonequivalent C-C bond lengths for metallic phase is only 0.006 Å [9].

Note that density functional theory (DFT) calculations for the (5,5) nanotube of a finite length [10,11] also gave a 60 atom periodicity of physical properties on the length of nanotube segment which is consistent with Kekule structure for infinite armchair nanotubes. Moreover, X-ray crystallographic analysis of chemically synthesized short (5,5) nanotubes [12] shows the Kekule bond length alternation pattern, which was in good agreement with DFT and PM3 calculations also performed in [12]. By the example of the infinite (5,5) nanotube, it was demonstrated that the structural Peierls transition connected with spontaneous symmetry breaking takes place not only with an increase of temperature, but also can be controlled by uniaxial deformation of armchair nanotubes [9].

A dynamical behaviour of molecules encapsulated inside nanotubes can correspond to the following regimes: oscillations about a fixed position and/or a fixed orientation of the molecule (regime A), hindered motion along the nanotube axis and/or rotation of the molecule (regime B) and free motion and/or rotation of the molecule (regime C). In the present Letter, we show the possibility of changes of the dynamical behaviour of molecules encapsulated inside armchair nanotubes as a result of the Peierls transition in the nanotube structure. In other words, these changes can include switching between the regimes A and B, switching between the regimes B and C, and the changes in diffusion coefficients corresponding to the hindered motion and/or rotation of the molecule (regime B). The considered changes are possible in the case where the regime B takes place for at least one phase of the nanotube, i.e. the temperature *T*_P _of the Peierls transition should correspond to the temperature range of the regime B (the hindered motion and/or rotation of the molecule). In other words, the temperature *T*_P _should be of the same order of magnitude (or a few orders of magnitude less) as energy barriers Δ*E *for motion and/or rotation of the molecule inside the nanotube at this phase. Note that inverse melting of motion and/or rotation of the molecule is possible, if the Peierls transition from the high- to low-temperature phase of the nanotube occurs with switching from the regime B to the regime C or switching from the regime A to the regime B.

Estimations showed that the Peierls transition temperature is *T*_P _≃ 1-15 K [4,5,8]. According to calculations [13,14], the barriers of the value close to this temperature range were obtained for rotation of the fullerene C_60 _inside the C_60_@C_240 _nanoparticle. It was also found that the changes of bond lengths of the fullerene C_240_, the outer shell of these nanoparticles, within 0.06 Å lead to an increase of the barriers for rotation by more than an order of magnitude [13,14]. The changes of the nanotube bond lengths caused by the Peierls distortions are of the same order of magnitude (about 0.03 Å for the (5,5) nanotube [9]). Note also that the size of an encapsulated molecule, and therefore, the nanotube radius cannot be too large, since the magnitude of the Peierls distortions decreases with an increase of the armchair nanotube radius [6].

Thus, taking into account the above considerations, we have chosen the smallest fullerene C_20 _and the magnetic endofullerene Fe@C_20 _to investigate changes in the dynamical behaviour of molecules inside nanotubes at the Peierls transition. It has been shown that the (8,8) nanotube is the smallest armchair carbon nanotube which can encapsulate the fullerene C_20 _[15]. A carbon nanotube with the fullerene C_20 _inside was also used as a model system to simulate a drug delivery via the nanotube [16].

This Letter is organized as follows: "Fullerene and nanotube structures" section presents the DFT calculations of the structure of the C_20 _and Fe@C_20 _fullerenes and the PM3 calculations of the structure of the (8,8) nanotube. "Fullerene-nanotube interaction" section presents the semiempirical calculations of the barriers for motion and rotation of the fullerenes inside the nanotube. The section that succeeds the latter is devoted to the dynamical behaviour of molecules inside the nanotubes. Our conclusions are summarized in the final section.

## Fullerene and nanotube structures

Structures of the C_20 _and Fe@C_20 _fullerenes have been calculated using the spin-polarized density functional theory implemented in NWChem 4.5 code [17] with the Becke-Lee-Yang-Parr exchange-correlation functional (B3LYP) [18,19]. Eighteen inner electrons of the iron atom are emulated with the help of the effective core potential - CRENBS ECP [20] (only 8 valence *s*-*d *electrons are taken into account explicitly). The 6-31G* basis set is used for describing electrons of the carbon atoms.

The ground state of the fullerene C_20 _is found to be a singlet state and has ***D***_2*h *_symmetry. The calculated energy of the triplet state of the fullerene C_20 _is found to be 64 meV greater than the energy of the ground state. The ground state of the endofullerene Fe@C_20 _is found to be a septet state and has ***C***_2*h *_symmetry.

**Figure 2:**
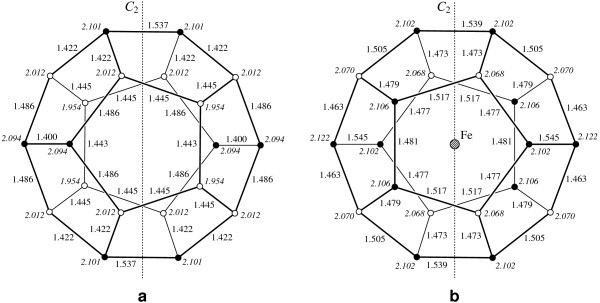
**Calculated structure of the C_20 _(a) and Fe@C_20 _(b) fullerenes**. The bond lengths are in ångströms. The distances between the carbon atoms and the fullerene centre (in ångströms) are denoted in italics. The atoms which have smaller and greater distances to the fullerene centre are shown by the open and filled circles, respectively. *C*_2 _symmetry axis is shown by the dotted line.

The calculated structures of the ground states of the C_20 _and Fe@C_20 _fullerenes are shown in Figure 2. The iron atom locates in the centre of the endofullerene. The smallest and the greatest distances between the carbon atoms and the C_20 _fullerene centre increase by 6 and 1%, respectively, as a result of the iron atom encapsulation.

The semiempirical method of molecular orbitals modified for one-dimensional periodic structures [21] with PM3 parameterization [22] of the Hamiltonian has been used to calculate the structure of the (8,8) nanotube. The method was used previously for calculating the Kekule structure of the (5,5) nanotube ground state and for studying structural transitions controlled by uniaxial deformation of this nanotube [9]. The adequacy of the PM3 parameterization of the Hamiltonian has been demonstrated [23] by the calculation of bond lengths of the C_60 _fullerene with ***I***_*h *_symmetry: the calculated values of the bond lengths agree with the measured ones [24] at the level of experimental accuracy of 10^-3 ^Å. The calculated Kekule structure of the (8,8) nanotube ground state is shown in Figure 1. The difference between the lengths of short and long bonds of this Kekule structure of the (8,8) nanotube is close to such a difference of the (5,5) nanotube [9]. The Peierls distortions include also radial distortions of the armchair carbon nanotube with periodicity of half of the translational period of the nanotube (for details see [9]). In the case of the (8,8) nanotube, the longest nanotube radius is 0.547 nm, while the shortest radius is 0.544 nm.

## Fullerene-nanotube interaction

The structures of the fullerenes C_20 _and Fe@C_20 _obtained in "Fullerene and nanotube structures" section have been used for finding the ground state position and for studying motion and rotation of these fullerenes inside the (8,8) nanotube. Two structures of the (8,8) nanotube have been considered: the Kekule structure calculated in "Fullerene and nanotube structures" section, and the structure with all equal bonds 1.423 Å in length (so that to be equal to the average bond length of the calculated the Kekule structure). The calculations of the interaction energy between walls of double-walled carbon nanotubes showed that the barriers for motion of the short wall relative to the long wall are very sensitive to the length of the long wall [25]. Thus, the size of the system is too large for *ab initio *calculations. The analogous problem exists also for the considered case of a single fullerene inside a nanotube. Therefore, the interaction between carbon atoms of the fullerenes and the nanotube at the interatomic distance *r *is described by the Lennard-Jones 12-6 potential(1)

with the parameters *ε *= 2.755 meV, *σ *= 3.452 Å. These parameters of the Lennard-Jones potential for the fullerene-nanotube interaction are obtained as the average values of the parameters [26] for fullerene-fullerene and fullerene-graphene interactions, in accordance with the procedure described in [26]. Here, the Lennard-Jones potential is used for calculating the potential surface of the interaction energy *E*_W _between the fullerene and the infinite nanotube, and we believe that this gives adequate qualitative characteristics of the potential surface shape. The cut-off distance, *r *= *r*_c _of the Lennard-Jones potential is taken equal to *r*_c _= 15 Å. For this cut-off distance the errors of calculation of the interaction energy *E*_W _between the fullerenes and the (8,8) nanotube and the barriers for relative motion and rotation of the fullerenes inside the nanotube are less than 0.1%. Both the fullerenes and the nanotube are considered to be rigid. An account of structure deformation is not essential for the shape of the potential surface both for the interwall interaction of carbon nanotubes [25,27] and the intershell interaction of carbon nanoparticles [13,14]. For example, the account of the structure deformation of the shells of C_60_@C_240 _nanoparticle gives rise to changes of the barriers for relative rotation of the shells which are less than 1% [13,14]. It should also be noted that the symmetry of interaction energy as a function of coordinates describing relative positions of interacting objects is determined unambiguously by symmetries of the isolated objects and does not change if the symmetries of the objects are broken because of their interactions.

The ground state interaction energies between the C_20 _and Fe@C_20 _fullerenes, and the (8,8) nanotube with Kekule structure are found to be -1.596 and -1.598 eV, respectively. The angles between the *C*_2 _symmetry axes of the C_20 _and Fe@C_20 _fullerenes and the nanotube axis at the ground states are 49.6° and 53.1°, respectively. The metastable states with the *C*_2 _symmetry axes of the fullerenes perpendicular to the nanotube axis are also found for both C_20 _and Fe@C_20_. At the metastable states, the interaction energies are greater by 4.58 and 3.04 meV than the ground state energies, for C_20 _and Fe@C_20_, respectively.

**Figure 3:**
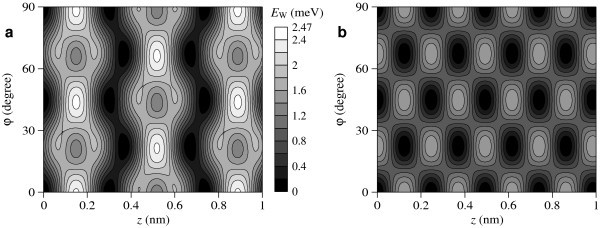
**The interaction energy *E*_W _(in meV) as a function of the fullerene C_20 _displacement *z *along the nanotube axis and the angle *φ *of the fullerene rotation about the nanotube axis**. **(a)** The (8,8) carbon nanotube with the Kekule structure; **(b)** the (8,8) carbon nanotube with the structure of metallic phase. The energy is given relative to the energy minima. The equipotential lines are drawn at an interval 0.2 meV.

**Figure 4:**
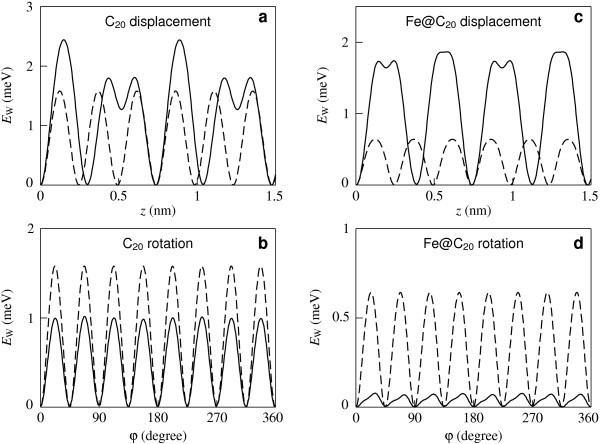
**Interaction energy between the C_20 _fullerene or Fe@C_20 _endofullerene and (8,8) nanotube**. Dependences of the interaction energy *E*_W _between the C_20 _fullerene **(a, b) **or Fe@C_20 _endofullerene **(c, d) **and the (8,8) nanotube on the fullerene displacement *z *along the nanotube axis **(a, c) **and on the angle *φ *of the fullerene rotation about the nanotube axis **(b, d)**. Solid lines denote nanotube with the Kekule structure, dashed lines denote nanotube with the structure of metallic phase. The energy minimum is positioned at *E*_W _= 0, *z *= 0 and *φ *= 0.

**Figure 5:**
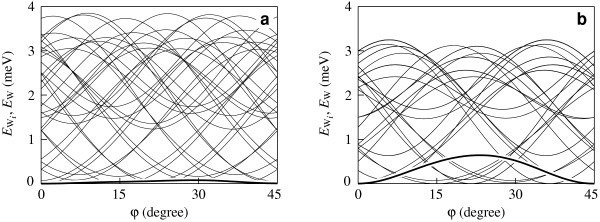
**Interaction energy between the Fe@C_20 _endofullerene and (8,8) nanotube**. Dependences of the interaction energy *E*_W*i *_between the Fe@C_20 _endofullerene and individual atoms of the (8,8) nanotube on the angle *φ *of rotation of the endofullerene about the nanotube axis are denoted by the thin lines. Dependence of the total interaction energy *E*_W _between the Fe@C_20 _endofullerene and the (8,8) nanotube on the angle *φ *is denoted by the thick line. **(a) **The (8,8) carbon nanotube with the Kekule structure; **(b) **the (8,8) carbon nanotube with the structure of metallic phase. All energies are given relative to the energy minima. Only dependences *E*_W*i *_with high values of the barriers Δ*E*_r*i *_are shown.

The potential surfaces of the interaction energy between the C_20 _fullerene and the nanotube, *E*_W_(*φ*, *z*), as functions of the relative displacement of the fullerene along the axis of the nanotube *z *and the angle of relative rotation of the fullerene about the axis of the nanotube *φ *are presented in Figure 3 for both the considered structures of the (8,8) nanotube. In the general case, diffusion of a fullerene along the nanotube axis is accompanied by rotation of the fullerene. Our calculations show that the barriers for rotation of both fullerenes about the axes which are perpendicular to the nanotube axis lie between 3 and 23 meV for any orientation of the fullerene and for both the considered structures of the (8,8) nanotube. These barriers are significantly greater than the barriers for rotation of the fullerenes about the axis of the nanotube (shown in Figure 3 for the C_20 _fullerene). Therefore, diffusion of the fullerenes along the nanotube axis is accompanied only by rotation about the nanotube axis. Thus, the minimal barrier Δ*E*_d _for diffusion of the fullerenes along the nanotube axis is the barrier between adjacent minima of the potential surface *E*_W_(*φ, z*). Figure 3 shows that the shapes of the potential surface *E*_W_(*φ*, *z*), corresponding to the Kekule structure of the (8,8) nanotube and to the structure of metallic phase are essentially different. For a case of the structure corresponding to the metallic phase of the (8,8) nanotube, all the barriers between adjacent minima of the potential surface *E*_W_(*φ, z*) are equivalent. In this case, the same barrier Δ*E*_d _= Δ*E*_r _should be overcome for diffusion of the fullerenes along the nanotube axis and for rotation of the fullerenes about this axis (see Figure 3b). For a case of the Kekule structure, two different barriers between the adjacent minima exist: the barrier Δ*E*_d _for diffusion of the fullerenes along the nanotube axis, and the barrier Δ*E*_r _to rotation of the fullerenes about this axis (see Figure 3a). For the Fe@C_20 _fullerene, the shapes of the potential surfaces *E*_W_(*φ, z*) are qualitatively the same as for C_20 _for both the considered structures of the nanotube. The calculated values of the barriers Δ*E*_d _and Δ*E*_r _are listed in Table 1 for both fullerenes. The dependences of the interaction energy between the C_20 _and Fe@C_20 _fullerenes and the nanotube on the relative displacement of the fullerene along the axis of the nanotube and the angle of its relative rotation about this axis, corresponding to both the considered structures of the (8,8) nanotube are compared as shown in Figure 4. Figure 4 is a vivid illustration of the significant changes of the barriers Δ*E*_d _and Δ*E*_r_. The most dramatic change corresponds to the rotation of the Fe@C_20 _fullerene about the nanotube axis. Let us discuss the reason of the significant changes of the barriers Δ*E*_d _and Δ*E*_r _at the Peierls transition by the example of the barrier Δ*E*_r _for rotation of the Fe@C_20 _endofullerene about the nanotube axis. Figure 5 presents the dependences *E*_W*i*_(*φ*) of the interaction energies between the endofullerene and individual atoms of the nanotube on the angle *φ *of rotation of the fullerene. Figure 5 shows that maxima of dependences *E*_W*i*_(*φ*) for individual atoms of the nanotube occur at different angles *φ*_m*i *_and so the dependence *E*_W_(*φ*) of total energy on the angle of rotation is essentially smoothed. In other words, the barrier Δ*E*_r _in the dependence *E*_W_(*φ*) of the *total *interaction energy between the endofullerene and the nanotube is less by an order of magnitude than the barriers Δ*E*_r*i *_in the dependences of the interaction energy between the endofullerene and *only one of the nanotube atoms*. Thus, the barrier Δ*E*_r _is very sensitive to the values of the barriers Δ*E*_r*i *_and angles *φ*_m*i*_. Therefore, the barrier Δ*E*_r _changes considerably at the found changes of the barriers Δ*E*_r*i *_and angles *φ*_m*i *_obtained from the Peierls distortions of the nanotube structure and the change of the nanotube symmetry at the transition. It should also be noted that small barriers to relative motion of nanoobjects resulting from the compensation of contributions of individual atoms to the barriers is a phenomenon well studied by the examples of such systems as double-shell carbon nanoparticles [13,14], double-walled carbon nanotubes [25,27-32] and a graphene flake in a graphite surface [33]. The considerable changes of barriers for relative rotation of shells at small changes of shell structure were found also for double-shell carbon nanoparticles [13,14].

**Table 1:** Calculated characteristics of the dynamical behaviour of the C_20 _and Fe@C_20 _fullerenes inside the (8,8) nanotube of different structure

Nanotube structure	Kekule structure		Structure of metallic phase	
**Fullerene**	**C_20_**	**Fe@C_20_**	**C_20_**	**Fe@C_20_**

Δ*E*_d _(meV)	1.68	1.73	0.87	0.44
Δ*E*_r _(meV)	0.33	0.05	0.87	0.44
*ν*_d _(GHz)	72.2	54.8	75.7	51.3
*ν_z _*(THz)	0.439	0.144	0.558	0.329
*ν_x _*(THz)	1.30	1.25	1.36	1.13
*ν_y _*(THz)	2.15	2.82	2.08	2.14

Δ*E*_d _is the barrier for diffusion of the fullerenes along the nanotube axis, Δ*E*_r _is the barrier for rotation of the fullerenes about the nanotube axis, *ν*_d _is the frequency of small relative vibrations of the fullerenes along the nanotube axis, *ν_z _*is the frequency of small relative rotational vibrations of the fullerenes about the nanotube axis, *ν_x _*and* ν_y _*are the frequencies of small relative rotational vibrations of the fullerenes about the two mutually perpendicular lateral axes.

The frequencies of small vibrations of the fullerenes along the nanotube axis (*ν*_d_), rotational vibrations about the nanotube axis (*ν_z_*) and rotational vibrations about two mutually perpendicular lateral axes (*ν_x_*, *ν_y_*) are also calculated and listed in Table 1. The most remarkable change of frequency as a result of the structural phase transition corresponds to rotational vibrations of the Fe@C_20 _fullerene about the nanotube axis (this agrees with the changes of the barriers).

## Dynamical behaviour of molecules inside nanotube

Let us consider the possible changes of the dynamical behaviour of the C_20 _and Fe@C_20 _fullerenes inside the (8,8) nanotube caused by the structural phase transition. The Peierls instability transition temperature *T*_P _was estimated for the (5,5) nanotube to correspond to temperature range *T*_P _≃ 1-15 K [4,5,8]. Both barriers Δ*E*_d _and Δ*E*_r _and the thermal energy *k*_B_*T*_P _are of the same order of magnitude at the structural Peierls phase transition (see Table 1). Therefore, dramatic changes of the diffusion and drift over these barriers can take place at the Peierls transition for the considered pairs of the encapsulated molecules and the nanotube.

Recently, the diffusion and drift in the periodic potential surface of the interaction energy dependent on the displacement *z *along the nanotube axis and the angle *φ *of the rotation about the nanotube axis were considered for the thread-like relative motion of walls of double-walled carbon nanotubes [28,29]. The expressions for the diffusion coefficient and mobility of a movable wall were obtained [28,29]. In this study, we consider specific cases of diffusion of the molecules along the nanotube axis and rotational diffusion about this axis. In this case, the expressions for diffusion coefficients, *D*_d _and *D*_r_, mentioned above and corresponding to the diffusion along the nanotube axis and rotational diffusion about this axis, respectively, take the form:(2)

where Ω_d _and Ω_r _are the pre-exponential multipliers in the Arrhenius formula for the frequency of jumps of the molecule between two neighbouring global minima of the potential surface *E*_W_(*φ*, *z*), *δ*_d _is the distance between neighbouring global minima for the motion of the molecule along the nanotube axis, *δ*_r _is the angle between neighbouring global minima corresponding to the molecule rotation about the nanotube axis and *k*_B _is the Boltzmann constant. The mobility *B*_d _for the motion along the axis can be easily obtained from the diffusion coefficient *D*_d _using the Einstein ratio *D*_d_/*B*_d _= *k*_B_*T*. Figure 3 shows that *δ*_d _= 0.123 nm and *δ*_d _= 0.37 nm for the (8,8) nanotube with the structure of the metallic phase and the Kekule structure, respectively, and *δ*_r _= 22.5° for the both structures of this nanotube.

The value of the pre-exponential multiplier Ω in the Arrhenius formula is usually considered to be related with the frequency *ν *of corresponding vibrations. We suppose that the ratio Ω*/ν *remains the same for relative motion of different carbon nanoobjects with graphene-like structure (nanotube walls and fullerenes). For reorientation of the fullerenes of the C_60_@C_240 _nanoparticle, the frequency multiplier Ω was estimated by molecular dynamics simulations having the value of 650 *± *350 GHz [13,14]. We expand the potential surface of the intershell interaction energy near the minimum using the same empirical potential as in [13,14], and calculate the frequencies of small relative librations of the shells. The calculated libration frequency has the value *ν *≈ 50 GHz, an order of magnitude less than that of the frequency multiplier Ω. In the estimations of this study, we use the values Ω_d _≈ 10*ν*_d _and Ω_r _≈ 10*ν_z _*for the pre-exponential multipliers.

**Figure 6:**
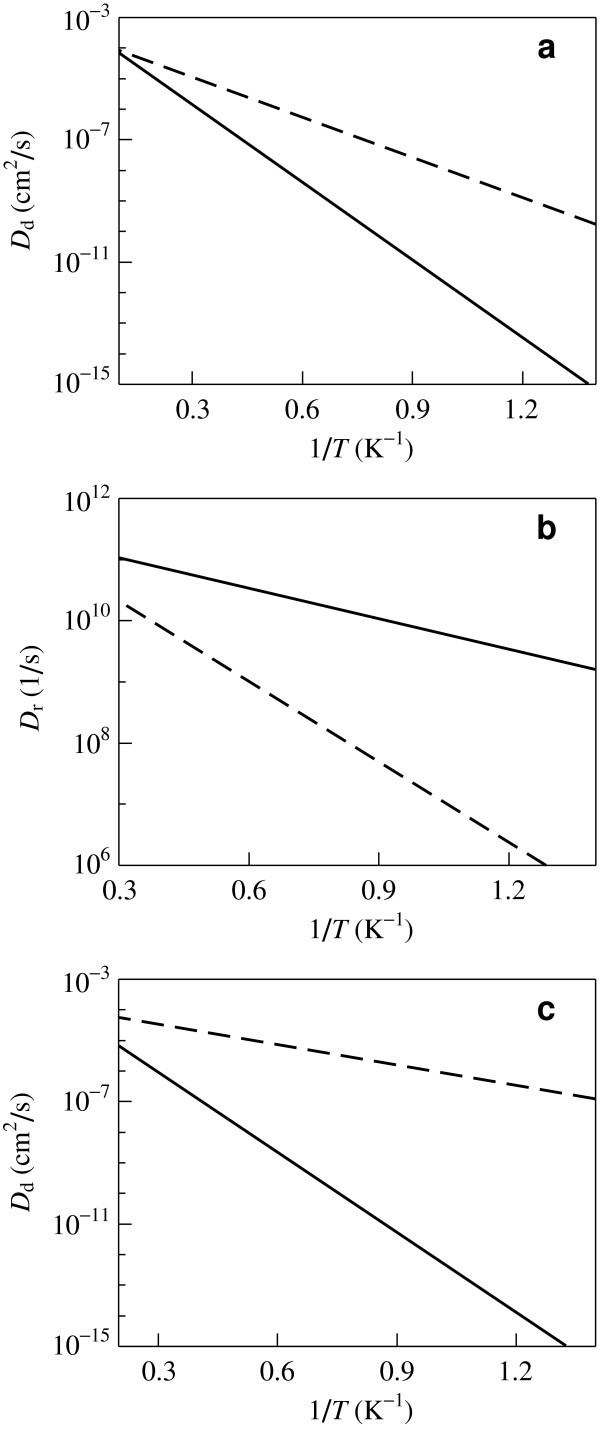
**Dependences of the diffusion coefficients on the reciprocal of temperature 1/*T***. **(a, c) **The diffusion coefficient *D*_d_; **(b) **the diffusion coefficient *D*_r_. **(a, b) **The C_20 _fullerene; **(c) **the Fe@C_20 _endofullerene. Solid lines: the (8,8) carbon nanotube with the Kekule structure; dashed lines: the (8,8) carbon nanotube with the structure of metallic phase.

The temperature dependencies of the diffusion coefficients, *D*_d _and *D*_r_, estimated using expressions (2) are shown in Figure 6. The dependence of the interaction energy *E*_W_(*z*) on the displacement of the fullerene C_20 _has two different barriers between the neighbour minima for the case of the nanotube with the Kekule structure (see Figure 4a). Since the frequency of the jumps of the fullerene between the neighbour minima exponentially depends on the barrier, the contribution of jumps over the lower barrier into the total diffusion coefficient is disregarded in these estimations. The temperature range corresponding to the Peierls transition temperature estimates *T*_P _≃ 1-15 K [4,5,8] and the cases Δ*E*_d_/*k*_B_*T <*1 and Δ*E*_r_/*k*_B_*T <*1, where the Arrhenius formula is adequate, are considered. (For rotation of the Fe@C_20 _inside the nanotube with the Kekule structure the Arrhenius formula is not applicable (Δ*E*_r_/*k*_B_*T >*1) at this temperature range; this case is considered below.) Figure 6 shows that the changes of the diffusion coefficients, *D*_d _and *D*_r_, at the Peierls transition can be of orders of magnitude. It is of interest that the diffusion coefficient *D*_r _for rotational diffusion of the C_20 _fullerene decreases at the Peierls transition with the increase of temperature.

If a molecule is encapsulated inside a nanotube without a structural phase transition, the jump rotational diffusion takes place at low temperatures, Δ*E*_r_/*k*_B_*T >*1, and the free rotation of the molecule occurs at high temperature, Δ*E*_r_/*k*_B_*T <*1. Orientational melting (a loss of the orientational order with an increase of temperature) has a crossover behaviour if the structural phase transition is absent. Firstly, orientational melting was considered for two-dimensional clusters with shell structure [34-37] and later for double-shell carbon nanoparticles [13,14], double-walled carbon nanotubes [25,38,39] and carbon nanotube bundles [40]. In the case where a molecule is encapsulated inside a nanotube with a structural phase transition and the barrier Δ*E*_r _for rotation of the molecule is greater for the high-temperature phase than for the low-temperature phase, an inverse orientational melting (a loss of the orientational order with a decrease of temperature) is possible. In other words, the inverse orientational melting takes place if the Peierls transition temperature lies in the range Δ*E*_rl _*< k*_B_*T*_P _*<*Δ*E*_rh_, where Δ*E*_rh _and Δ*E*_rl _are the barriers for molecule rotation corresponding to high-temperature and low-temperature phases of the nanotube, respectively. For the considered molecules inside the (8,8) nanotube, these temperature ranges are estimated to be 3.8 *< T*_P _(K) *<*10 and 0.58 *< T*_P _(K) *<*5.1 for the C_20 _and Fe@C_20 _fullerenes, respectively (see Table 1). As these temperature ranges are in agreement with the Peierls transition temperature estimates *T*_P _≃ 1-15 K [4,5,8], we predict that the inverse orientational melting is possible for the systems considered. The inverse orientational melting should be more prominent for the case of the Fe@C_20 _fullerene with the greater ratio of the barriers Δ*E*_rh_/Δ*E*_rl_.

Let us discuss the possibility of observing the changes of the dynamical behaviour of molecules inside armchair carbon nanotubes at the Peierls transition. We believe that the most promising method is high-resolution transmission electron microscopy. This method was used for visualizing dynamics of processes inside nanotubes, such as reactions of fullerene dimerization with monitoring of time-dependent changes in the atomic positions [41] and rotation of fullerene chains [42]. The rotational dynamics of C_60 _fullerenes inside carbon nanotube was studied also by analysing the intermediate frequency mode lattice vibrations using near-infrared Raman spectroscopy [43]. The orientational melting in a single nanoparticle may be revealed also by IR or Raman study of the temperature dependence of width of spectral lines. A specific heat anomaly in multiwalled carbon nanotubes may be caused by the orientational order-disorder transition [44]. In the case of encapsulated magnetic molecules (for example, the Fe@C_20 _endofullerene considered above), the study of the temperature dependence of the electron spin resonance spectra could yield information on the molecule rotational dynamics of these molecules [45].

## Conclusive remarks

In this letter, we consider the changes of dynamical behaviour of fullerenes encapsulated in armchair carbon nanotubes caused by the Peierls transition in the nanotube structure by the example of the C_20 _and Fe@C_20 _fullerenes inside the (8,8) nanotube. We apply the DFT approach to calculate the structure of the C_20 _and Fe@C_20 _fullerenes. The ground state of the (8,8) nanotube is found to be the Kekule structure using the method of molecular orbitals. The Lennard-Jones potential is used for calculating the barriers for motion of the fullerenes along the axis and rotation about the axis of the (8,8) nanotube with the Kekule structure and the structure with all equal bonds corresponding to low-temperature and high-temperature phases, respectively. We show that the changes in the coefficients of diffusion of the fullerenes along the nanotube axis and their rotational diffusion at the Peierls transition can be as much as several orders of magnitude. The possibility of the inverse orientational melting at the Peierls transition is predicted. The analogous changes of dynamical behaviour are also possible for other large molecules inside armchair nanotubes. We believe that the predicted dynamical phenomena can be observed using high-resolution transmission electron microscopy, near-infrared Raman spectroscopy, specific heat measurements, and by study of electron spin resonance spectra for magnetic molecules.

## Abbreviation

DFT: density functional theory.

## Competing interests

The authors declare that they have no competing interests.

## Authors' contributions

NAP and NAV set the problem of dynamical behaviour of small fullerenes inside carbon nanotubes. NAP, SAV, EFK, NNH and ONB performed the calculations of the fullerene and nanotube structures. NAP, NAV and EFK contributed to the analysis of the fullerene and nanotube structures. IVL, AAK, SAV and ONB performed the calculations of the interaction between the fullerenes and the nanotube. AMP and YEL proposed the idea of inverse orientational melting. AMP, YEL, NAP and EFK contributed to the discussion of the dynamical behaviour of the fullerenes inside the nanotube and wrote the original manuscript. All authors commented on the manuscript and approved the final manuscript.
